# Inappropriate Telemetry Use Is Increased during the COVID-19 Era

**DOI:** 10.3390/healthcare9121610

**Published:** 2021-11-23

**Authors:** Jean Kim, Kyle Miyazaki, Yoshito Nishimura, Ryan Honda

**Affiliations:** Department of Internal Medicine, University of Hawai‘i, Honolulu, HI 96813, USA; kylesmiy@hawaii.edu (K.M.); nishimura-yoshito@okayama-u.ac.jp (Y.N.); ryhonda@queens.org (R.H.)

**Keywords:** telemetry, cardiac telemetry, COVID-19, quality improvement, hospitalist, hospital medicine, Choosing Wisely Campaign

## Abstract

Due to the unprecedented COVID-19 pandemic, there may be overuse of telemetry monitoring compared to the pre-pandemic period. We compared the frequency of inappropriate telemetry use in the pre-COVID-19 period (1 November 2019 to 28 February 2020) versus the peri-COVID-19 period (1 March 2020 to 30 June 2020) at a major academic hospital in Honolulu, Hawaii, by a retrospective chart review to assess for the appropriateness of the telemetry orders during this period, based on the 2017 American College of Cardiology/American Heart Association guidelines. Compared to the pre-COVID-19 period, there was a significant increase in inappropriate telemetry use during the peri-COVID-19 period (X2 (1, *N* = 11,727) = 6.59, *p* = 0.0103). However, there was no increase in the proportions of respiratory failure (4.0%) or pneumonia (2.7%) during the peri-COVID-19 period. The increase in inappropriate telemetry use may be related to the uncertainty in clinical care and decision making amid the pandemic of the new virus. Appropriate utilization of telemetry monitoring is increasingly important during the pandemic due to the limited availability of resources. Further investigation is needed to clarify the relationship between the pandemic and trends in telemetry ordering.

## 1. Introduction

Although the electrocardiogram (ECG) was invented more than 100 years ago, the concept of continuous cardiac monitoring was not available until the early 1960s [1]. Telemetry is a frequently utilized and essential modality of cardiac monitoring in acute hospitalization settings for rhythm surveillance and diagnosis of arrhythmias. However, it can be associated with high costs and the utilization of resources [2].

In order to guide its appropriate use, practice guidelines were implemented in 2004 with an update in 2017 by the American College of Cardiology/American Heart Association (ACC/AHA) [3]. The use of telemetry remains widespread; studies have demonstrated that as many as 43% of monitored patients continue to receive telemetric monitoring despite a lack of appropriate indications [4]. In fact, inappropriate continuous telemetry monitoring was identified as its top five focus of the Choosing Wisely campaign [5]. Familiarizing oneself with telemetry indications and interpreting telemetry data is crucial for physicians both from a clinical as well as a high-value care standpoint.

In addition, the coronavirus 2019 (COVID-19) pandemic has been known for its impact on both the respiratory and cardiovascular systems in the setting of systemic inflammation [6]. However, the benefits of telemetry monitoring of COVID-19 patients are still unclear. Rather, it may lead to more harm associated with the increased chance of contact related to cardiac monitoring. In this study, we compare the frequency of inappropriate telemetry ordering in the pre-COVID-19 pandemic period and during the COVID-19 pandemic at the Queen’s Medical Center, a major academic medical center in Hawaii.

## 2. Materials and Methods

### 2.1. Study Design and Definitions

A retrospective review of the electronic medical record was performed for all of the cardiac telemetry orders at the Queen’s Medical Center in Honolulu, Hawaii, between 1 November 2019 through 30 June 2020. The study was divided further into the “Pre-COVID-19” and the “Peri-COVID-19” periods to distinguish the cases between 1 November 2019 through 28 February 2020, and 1 March 2020 through 30 June 2020, respectively. These period dates were chosen in accordance with the World Health Organization (WHO)’s declaration of the COVID-19 pandemic that took place on 11 March 2020.

During the pre-COVID-19 period, there were a total of 6168 telemetry and 5559 telemetry orders during the pre-COVID-19 period and the peri-COVID-19 period, respectively. All telemetry orders were reviewed for their reason of order, including the miscellaneous comments provided by the ordering physicians explaining their clinical reasons for telemetry requests. The telemetry orders were assessed for their appropriateness in accordance with the 2017 American College of Cardiology (ACC)/American Heart Association (AHA) guidelines for electrocardiographic monitoring in hospital settings [3]. Telemetry indications with Class IA and Class IB level of evidence were considered “appropriate” indications (Supplementary Table S1). All of the others were determined to be “inappropriate” reasons for telemetry (Table 1). It should be noted that although the 2017 ACC/AHA guidelines recommend initial telemetry monitoring for electrolyte abnormalities involving moderate to severe imbalances of potassium or magnesium (Class I; Level of Evidence B), the guidelines do not identify discrete values defining the “moderate to severe” electrolyte disturbances. As such, in this study, all of the telemetry orders attributed to electrolyte disturbances were considered to be “inappropriate”.

### 2.2. Statistical Analysis

The chi-square test was performed to compare the frequency of inappropriate telemetry orders between the previously defined pre-COVID and the COVID-19 periods. The threshold for significance was defined as the *p*-value < 0.05. All of the statistical analyses were conducted with JMP Version 15.1 (SAS Institute, Cary, NC, USA).

## 3. Results

### 3.1. Characteristics of Inappropriate Telemetry Use

Table 1 summarizes the information about telemetry ordering, including common reasons for inappropriate telemetry orders in the pre-COVID and the COVID periods. During the pre-COVID-19 period, 533/6168 telemetry orders were noted to be inappropriate (8.6%). During the peri-COVID-19 period, 557/5559 orders were inappropriate (10.0%). Compared to the pre-COVID-19 period, there was a significant increase of inappropriate telemetry ordering during peri-COVID-19 period (X2 (1, *N* = 11,727) = 6.59, *p* = 0.0103).

### 3.2. Reasons for Inappropriate Telemetry Ordering during the Pre- and Peri-COVID-19 Periods

**Figure 1:**
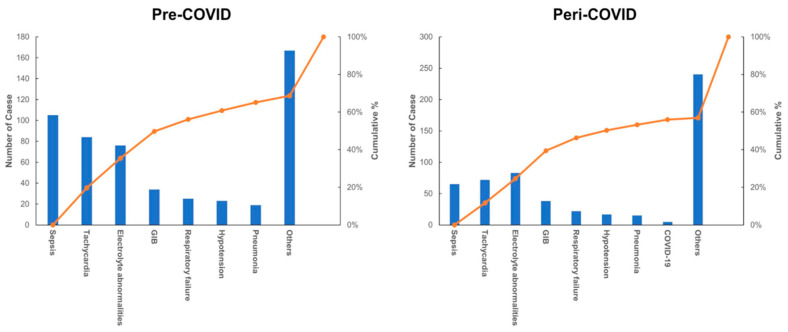
A Pareto chart depicting the major reasons for inappropriate telemetry ordering during the pre-COVID-19 and the peri-COVID-19 periods.

The main reasons for inappropriate telemetry reasons are summarized in Table 1 and Figure 1. During the pre-COVID-19 period, common reason for inappropriate telemetry order included sepsis (*n* = 105, 19.7% of the total inappropriate telemetry ordering), tachycardia (*n* = 84, 15.8%), electrolyte abnormalities (*n* = 76, 14.2%), and gastrointestinal bleeding (*n* = 34, 6.4%). Among them, respiratory failure and pneumonia accounted for 4.7% and 3.6%, respectively. During the peri-COVID-19 period, sepsis, tachycardia, and electrolyte abnormalities continued to account for the majority of inappropriate telemetry ordering. There was no increase in the proportions of respiratory failure (4.0%) or pneumonia (2.7%) during the peri-COVID-19 period. Only five cases (0.9%) of inappropriate telemetry ordering reason with “COVID-19” were noted. The reasons for inappropriate telemetry ordering noted as “Others” were widely varied as they were manually entered in by the ordering providers, and the miscellaneous reasons included seizures, altered mental status, generalized weakness, renal failure, and anemia.

## 4. Discussion

To the best of our knowledge, the present study is the first observational study comparing the frequency of inappropriate telemetry use during pre-COVID-19 and peri-COVID-19 periods. Our results underscore the significant increase in inappropriate telemetry orders during the peri-COVID-19 period compared to the pre-COVID-19 period. Interestingly, however, inappropriate telemetry orders related to COVID-19 and the related conditions such as respiratory failure comprise only a small portion of the inappropriate orders. Although it is unclear why the number of inappropriate telemetry orders was increased during the latter period, it could be related to the increased uncertainty in clinical care and decision making and lack of solid guidelines amid the pandemic of the new virus. Other speculation includes the misconception of closer monitoring or improved outcomes for low-risk patients under telemetry; lack of awareness of the practice standards for utilizing telemetry; or decisions made based on weighing the risks of patients entering the hospital setting that may subject them to become vulnerable to the virus.

While several articles reported that cardiac monitoring might be helpful to detect occult arrhythmias or prolongation of QT interval in COVID-19 patients [7,8,9,10,11], most of the studies were performed during the early phase of the pandemic when hydroxychloroquine and azithromycin, medications with the notorious side effect of QT interval prolongation, were still considered effective for COVID-19 infection. Thus, it is still unclear how beneficial telemetry monitoring may be for the management of COVID-19 patients.

Telemetry can potentially improve overall outcomes in the appropriate patient population by continuously monitoring cardiac rate and rhythm and detecting lethal arrhythmias or unwitnessed cardiac arrest. Inappropriate telemetry use has unfavorable consequences, however, including increased unnecessary diagnostic or therapeutic interventions, workflow interruptions, alarm fatigue, and a rise in healthcare cost [12]. Of note, the cost of daily telemetry monitoring, which is estimated to be up to USD 1400 per patient, raises a major concern associated with inappropriate telemetry [13,14]. As the Society of Hospital Medicine recommended in its Choosing Wisely campaign, a continuous telemetry monitoring outside the intensive care unit is not recommended without appropriate reasons. Even during the pandemic, appropriate utilization of healthcare resources, including telemetry, needs to be emphasized.

Several limitations in this study should be noted. First, the study period includes the early phase of the COVID-19 pandemic through 30 June 2020. A follow-up study may be helpful to better characterize the frequency and the reasons for inappropriate telemetry orders in the setting of the ongoing pandemic. Secondly, this was a single-institution study and inappropriate telemetry orders in our hospital accounted for only approximately 10%, which is considerably lower than that of other studies (18–43%) [4] and may affect the generalizability of the study results. The lower frequency of inappropriate telemetry use may have been because of our institution’s recent changes in the system that mandates ordering providers to choose from or manually enter in the ordering reasons for telemetry and also automatic reminders in 24 and 48 h to review the reasons for telemetry again. Despite these limitations, the study presents important evidence that the pandemic may increase the frequency of inappropriate telemetry use, which leads to alarm fatigue and incurs unnecessary healthcare costs. Further investigation and a follow-up study are crucial to clarify the relationship between the pandemic and trends in telemetry ordering.

## 5. Conclusions

In conclusion, our study has shown the increased frequency of inappropriate telemetry use during the COVID-19 period compared to the pre-COVID-19 period. Further, larger-scale study with an extended follow-up period is needed to corroborate our findings and to elucidate reasons behind this finding.

## Figures and Tables

**Table 1:** The list of inappropriate telemetry order reasons and their frequency during the pre-COVID-19 and the peri-COVID-19 periods.

Telemetry Order Reasons	Pre-COVID-19 Period (1 November 2019–28 February 2020) Case (%)	Peri-COVID-19 Period (1 March 2020–30 June 2020) Case (%)	*p*-Value
Total Telemetry Cases	6168	5559	
Appropriate	5635 (91.4)	5002 (90.0)	0.0103 *
Inappropriate	533 (8.6)	557 (10.0)	
Reasons for Inappropriate Ordering			
Sepsis	105 (19.7)	65 (11.7)	
Tachycardia	84 (15.8)	72 (12.9)	
Electrolyte abnormalities	76 (14.2)	83 (14.9)	
GIB	34 (6.4)	38 (6.8)	
Respiratory failure	25 (4.7)	22 (4.0)	
Hypotension	23 (4.3)	17 (3.0)	
Pneumonia	19 (3.6)	15 (2.7)	
COVID-19	N/A	5 (0.9)	
Others	167 (31.3)	240 (43.1)	

Abbreviations: COVID-19, coronavirus disease 2019; GIB, gastrointestinal bleeding; N/A, not applicable. * Statistically significant difference in the frequency of inappropriate tele use pre- and peri-COVID-19 periods (*p* < 0.05). X^2^ (1, *N* = 11,727) = 6.59, *p* = 0.0103.

## Data Availability

The datasets generated and analyzed during the current study are available from the corresponding author upon reasonable request.

## Supplementary Materials

The following are available online at https://www.mdpi.com/article/10.3390/healthcare9121610/s1, Table S1: Recommended indications of electrocardiographic monitoring for hospitalized patients.
