# Supplementary material for: Inappropriate Telemetry Use Is Increased during the COVID-19 Era
Source: Healthcare (Basel). 2021 Nov 23;9(12):1610. doi: 10.3390/healthcare9121610 (PMC8700992; doi:10.3390/healthcare9121610)
Supplement: Supplementary file 1 [file healthcare-09-01610-s001.zip › healthcare-1456165-supplementary.pdf]

Table S1. Recommended Appropriate Indications of Electrocardiographic Monitoring for Hospitalized.

**Patient Population/Indication**	**Level of Evidence ***
Early-phase ACS (<24h) for intermediate or high-risk NSTE-ACS or STEMI	*Class I; Level of Evidence B*
Post-MI, with revascularization of all ischemic lesions	*Class I; Level of Evidence B*
Post-MI, without revascularization of all ischemic lesions	*Class I; Level of Evidence C*
Following open heart surgery	*Class I; Level of Evidence B*
Syncope of suspected cardiac origin	*Class I; Level of Evidence B*
Targeted temperature management	*Class I; Level of Evidence C*
Clinically significant cardiovascular or hemodynamic deterioration	*Class I; Level of Evidence C*
VT, post-resuscitation or hemodynamically unstable	*Class I; Level of Evidence C*
Atrial tachyarrhythmia (new or recurrent AF, hemodynamically unstable, or not rate controlled)	*Class I; Level of Evidence C*
Symptomatic sinus bradycardia and/or second- or third-degree AV block	*Class I; Level of Evidence C*
Transcutaneous pacing pad	*Class I; Level of Evidence C*
ICD shocks requiring hospital admission	*Class I; Level of Evidence C*
Acute decompensated heart failure	*Class I; Level of Evidence B*

Abbreviations: ACS, acute coronary syndrome; AF, atrial fibrillation; AV, atrioventricular; ICD, implantable; NSTE, Non-ST-elevated; STEMI, ST-elevated myocardial infarction; MI, myocardial infarction; VT, ventricular tachycardia; * Level of Evidence as published in 2017 ACC/AHA Update to Practice Standards for Electrocardiographic Monitoring in Hospital Settings [3].
